# Emerging reporting and verification needs under the Paris Agreement: How can the research community effectively contribute?

**DOI:** 10.1016/j.envsci.2021.04.012

**Published:** 2021-08

**Authors:** Lucia Perugini, Guido Pellis, Giacomo Grassi, Philippe Ciais, Han Dolman, Joanna I. House, Glen P. Peters, Pete Smith, Dirk Günther, Philippe Peylin

**Affiliations:** a Foundation Euro-Mediterranean Center on Climate Change (CMCC), Division on Climate Change Impacts on Agriculture, Forests and Ecosystem Services (IAFES), Viale Trieste n. 127, 01100, Viterbo, Italy; b European Commission, Joint Research Centre, Directorate for Sustainable Resources, Via Enrico Fermi n. 2749, 21027, Ispra, VA, Italy; c Laboratoire des Sciences du Climat et de l’Environnement, (LSCE) CEA CNRS UVSQ UPSACLAY, 91191, Gif-sur-Yvette, France; d Vrije Universiteit Amsterdam, Department of Earth Sciences, Faculty of Science, Boelelaan 1085, Amsterdam, the Netherlands; e University of Bristol, School of Geographical Science, University Road, BS8 1SS, Bristol, UK; f CICERO Center of International Climate Research, Pb. 1129 Blindern, 0318, Oslo, Norway; g University of Aberdeen, Institute of Biological and Environmental Sciences, 23 St Machar Drive, AB24 3UU, Aberdeen, UK; h Umweltbundesamt / German Environment Agency, Postfach 1406, 06813, Dessau-Roßlau, Germany

**Keywords:** Climate change, Research contribution, Developing countries, GHG inventory, Emission estimates, Atmospheric observations

## Abstract

Greenhouse gas (GHG) emission inventories represent the link between national and international political actions on climate change, and climate and environmental sciences. Inventory agencies need to include, in national GHG inventories, emission and removal estimates based on scientific data following specific reporting guidance under the United Nation Framework Convention on Climate Change (UNFCCC) and the Paris Agreement, using the methodologies defined in the Intergovernmental Panel on Climate Change (IPCC) Guidelines. Often however, research communities and inventory agencies have approached the problem of climate change from different angles and by using terminologies, metrics, rules and approaches that do not always match. This is particularly true dealing with “Land Use, Land-Use Change and Forestry” (LULUCF), the most challenging among the inventory sectors to deal with, mainly because of high level of complexity of its carbon dynamics and the difficulties in disaggregating the fluxes between those caused by natural and anthropogenic processes.

In this paper, we facilitate the understanding by research communities of the current (UNFCCC) and future (under the Paris Agreement) reporting requirements, and we identify the main issues and topics that should be considered when targeting improvement of the GHG inventory.

In relation to these topics, we describe where and how the research community can contribute to producing useful inputs, data, methods and solutions for inventory agencies and policy makers, on the basis of available literature. However, a greater effort by both communities is desirable for closer cooperation and collaboration, for data sharing and the understanding of respective and common aims.

## Highlights

• The research community will play a key role in the post-2020 UNFCCC framework.
• GHG inventories must follow a rigid set of rules established by the UNFCCC and IPCC.
• To be policy relevant, the research community should understand these set of rules.
• The research and reporting communities should facilitate estimates comparability.

## 1 Introduction

“If you can't measure it, you can't improve it”. This concept is also true within the context of climate policy, where the achievement of the objectives of the United Nations Framework Convention on Climate Change (UNFCCC) is dependent on the ability of the international community to accurately measure greenhouse gas (GHG) emission trends and, consequently, to alter these trends.

The reporting of GHG emissions under the UNFCCC aims to provide transparent, accurate, complete, consistent and comparable GHG estimates through national inventories. These national GHG inventories represent essential links between science and policy-making, providing fundamental data to inform domestic and global actions on climate change (Swart et al., 2007; Damassa and Elsayed, 2013; Pulles, 2017).

To achieve the provision of reliable and consistent GHG information, the Conference of the Parties (COP) has established a set of requirements for reporting annual national GHG inventories for developed country Parties (dec. 3/CP.5 – UNFCCC, 2000; dec. 24/CP.19 – UNFCCC, 2014), to be fulfilled in accordance with Intergovernmental Panel on Climate Change (IPCC) guidelines and guidance (IPCC, 2006), as adopted by the UNFCCC. Since the early 2000s, these countries have acquired extensive experience in measuring, reporting and verifying their GHG emissions and removals. However, there are still some gases (e.g., CH_4_, N_2_O) with high uncertainty levels in the inventories (Choi et al., 2017) and sectors (e.g., waste and agriculture) where countries use a low level of complexity and accuracy in the estimation methods (EEA, 2019). The Paris Agreement (UNFCCC, 2015) extended further the reporting obligations: its Enhanced Transparency Framework requires all Parties (including developing ones) to report their GHG balance on a biannual basis, and to track the progress of individual countries towards achieving their mitigation targets. While developed countries will continue to report estimates annually, these new requirements increase the need for tools and data for developing countries that, in many cases, do not yet have in place the necessary capacity to produce regular GHG inventories (Kawanishi and Fujikura, 2018).

The research community that is directly or indirectly involved in the estimation of GHG emissions and removals from natural and anthropogenic processes is expected to play an increasingly crucial role in the GHG reporting and verification work under the UNFCCC, and overall, in increasing confidence in GHG estimates. This research community, which includes public and private research centres, and national and international collaborative programs, already provides data and methods for GHG estimates and their verification at country level, and it will contribute to assessing the collective progress of countries in the context of the so-called Global Stocktake. The Global Stocktake is the periodical assessment (every 5 years, with the first on 2023) of the collective progress towards achieving the purpose of the Paris Agreement and its long-term goals related to mitigation, adaptation and means of implementation, on the basis of the best available science (dec. 19/CMA.1, par. 14 – UNFCCC, 2019b). The process aims at informing Parties whether their cumulated effort is on track to deliver the “well-below 2 °C” trajectory, thus providing an indication on the need to enhance actions through the update of their successive National Determined Contributions (NDCs) under the Paris Agreement. Although not all NDCs contain quantitative targets based on GHG emissions, ultimately inventories represent the foundation for tracking progress towards mitigation goals at global level.

In support of countries’ GHG reporting activities and policy decisions, a wide variety of research-based approaches have been provided, from remote sensing (e.g., Harris et al., 2012; Achard et al., 2014; Baccini et al., 2017), atmospheric and land-based *in-situ* data, and models aimed at providing GHG budgets at scales ranging from globe (e.g., Kirschke et al., 2013; Sitch et al., 2015; Houweling et al., 2015; Friedlingstein et al., 2019; Thompson et al., 2019; Harris et al., 2021; Hong et al., 2021) to large regions and countries (e.g., Gurney et al., 2002; Bergamaschi et al., 2015; Turner et al., 2015; Ganesan et al., 2017; Bergamaschi et al., 2018a), and for individual components of ecosystem fluxes, such as soil carbon (e.g., Smith et al., 2020). Moreover, several research approaches attempted to estimate GHG budgets of subnational territories (e.g., Lauvaux et al., 2008; Levin et al., 2011; Schuh et al., 2013; Wecht et al., 2014), cities (e.g., Kort et al., 2012; Bréon et al., 2015; Lauvaux et al., 2016; Broquet et al., 2018) or industrial sites/power plants (Nassar et al., 2017). While these scientific contributions play important roles in improving the collective knowledge on GHG fluxes and their measurement at local, national and global levels, in many cases they cannot be directly used for national GHG inventory purposes. This is because these approaches do not necessarily match the specific requirements that national inventory agencies need to follow under the UNFCCC, Paris Agreement and adopted IPCC guidelines. Therefore, an effective contribution by the research community to countries’ GHG reporting and verification efforts requires a full understanding of the current rules and the emerging needs under the Paris Agreement. These topics have been discussed in the context of the EU Horizon 2020 research project “Observation-based system for monitoring and verification of greenhouse gases” (VERIFY^1^). Through the project, several exchanges between the research community and GHG inventory agencies took place, highlighting a differing understanding of the purposes, outputs, languages, terminology and system boundaries used by these two communities involved in GHG emission/removal estimates.

This paper aims to build bridges between research community and inventory agencies. Specifically, it provides an overview of the current and future GHG reporting and verification requirements under the Paris Agreement, including the IPCC methods (in the supplementary materials), identifying how and where the research community can provide an effective contribution to support GHG inventory agencies and, therefore, towards the implementation of the Paris Agreement.

## 2 Current and future GHG inventory requirements

### 2.1 Pre-2020 framework

Based on the concept of “*common but differentiated responsibilities and respective capabilities*” (art.3.1 – UNFCCC, 1992), the UNFCCC introduced a broad differentiation between Parties listed in the Annex I of the Convention (essentially developed countries) and Parties not included in Annex I (mainly developing countries). Historically, under the Convention and its Kyoto Protocol (which covers the period 2008–2020), Annex I Parties have assumed the commitments to reduce their GHG emissions, and to provide financial support and technology transfer to Non-Annex I Parties, which did not have such commitments. According to decision 24/CP.19, the COP has established that Annex I Parties are mandated to report anthropogenic GHG emission and removal estimates annually, from 1990 to the current year minus 2 (time series), using the 2006 IPCC Guidelines for National Greenhouse Gas Inventories (hereafter called 2006 IPCC Guidelines). Differently, the Non-Annex I Parties are required, in a less prescriptive manner, to provide only summary GHG emissions information for specific years through their Biennial Update Reports. For Non-Annex I Parties, a comprehensive time series is not mandatory, and they are also allowed to use the older 1996 IPCC Guidelines, if they wish (dec. 2/CP.17, annex III – UNFCCC, 2012). A further level of flexibility is granted to the Least Developed Countries and to Small Island Developing States, recognising their specific circumstances related to vulnerability and adaptation to climate change (dec. 2/CP.17, par. 58b – UNFCCC, 2012).

The GHG inventories of Annex I Parties undergo independent annual reviews, where third party experts, from a roster of experts at the UNFCCC, check that the UNFCCC and IPCC guidelines are followed.

### 2.2 Post-2020 framework

The Paris Agreement (UNFCCC, 2015) has established a collective objective, applicable to all signatory Parties, that aims at holding “[…] *the increase in the global average temperature to well below 2 °C above pre-industrial levels and pursuing efforts to limit the temperature increase to 1.5 °C*” (article 2.1a), and a long term goal to reach a balance between emissions and removals of anthropogenic GHG in the second half of the 21st century (article 4.1). The Paris Agreement removes the previous distinction between Annex I Parties and Non-Annex I Parties in terms of targets and reporting, although recognising their different capacities and providing some flexibility to developing country Parties in specific fields of reporting (dec. 18/CMA.1, par. 4–6 of the annex – UNFCCC, 2019a).

The achievement of the Paris Agreement objectives is based on a bottom-up approach, where Parties submit their country’s efforts through their NDC, thus allowing each country to define their targets based on their national circumstances. The backbone of the Agreement is the Enhanced Transparency Framework, which comprises of a set of modalities, procedures and guidelines to estimate and report countries’ GHG fluxes, to track their progress towards achieving their individual NDC targets, and to provide guidance on the kind of information that should be included in the NDC (dec. 18/CMA.1 – UNFCCC, 2019a). The framework strongly builds on the previous UNFCCC arrangements, but the new requirements will supersede the previous arrangements, starting from the first reporting year (2024). The overall objective of the Enhanced Transparency Framework is to build trust and confidence among parties and the general public on the effectiveness of the undertaken actions. The framework is also key to inform the Global Stocktake in terms of GHG emissions levels, reductions achieved and future trajectories of foreseen actions under the NDC.

The modalities, procedures and guidelines of the Paris’ transparency framework are defined in the Katowice Rulebook, that requires all Parties to provide, on a biennial basis and starting from 2024, a Biennial Transparency Report (BTR - dec. 18/CMA.1, par.10 of the annex – UNFCCC, 2019a). Among other information, the BTR includes the national inventory report of anthropogenic emissions and removals, consisting of a national inventory document (including a description of the methods used), and common reporting tables, noting that developed countries still have to provide flux estimates annually, i.e., at the same frequency and level of detail as done in the past.

An advantage of the post-2020 modalities and procedures of the Enhanced Transparency Framework is the harmonization of the use of guidelines for all Parties. The Katowice Rulebook establishes that the use of the 2006 IPCC Guidelines is obligatory for all Parties from 2024 (dec. 18/CMA.1, par. 20 of the annex – UNFCCC, 2019a), as well as the use of common reporting and tabular formats, although their contents are still under discussion within the negotiation process (Rocha and Ellis, 2020). Meanwhile, IPCC approved the “2013 Supplement to the 2006 IPCC Guidelines for National Greenhouse Gas Inventories: Wetlands” and the “2019 Refinement to the 2006 IPCC Guidelines for National Greenhouse Gas Inventories” (hereafter called 2019 Refinement). These publications do not revise the 2006 IPCC Guidelines, but update, supplement and/or elaborate the 2006 IPCC Guidelines where gaps, new methodologies and new data have been identified. At present, neither the 2013 Supplement for Wetlands nor the 2019 Refinement are compulsory in GHG inventories as the first is only recommended (dec. 18/CMA.1, par. 20 of the annex – UNFCCC, 2019a) and the latter needs to be formally adopted under the Paris Agreement. However, Parties can use the 2019 Refinement in their inventories on a voluntary basis, as a source of updated scientific information.

Finally, the Parties will be subjected to a review process, called Technical Expert Review (article 13.11 – UNFCCC, 2015), which is the second main pillar of the Enhanced Transparency Framework. Regarding the mitigation aspects, this consists in a facilitative process aimed at checking that the information included in the parties' national GHG inventories and those necessary to track and achieve their NDC are consistent with the Katowice Rulebook modalities, procedures and IPCC guidelines (dec. 18/CMA.1, par. 146 of the annex – UNFCCC, 2019a). Consequently, the inventory agencies have well defined margins of manoeuvre in terms of type of data, timing of submission, methodologies and approaches that they can use in their inventories. Therefore, it is essential that the research community becomes familiar with them and that it fully understands the main elements to be fully supportive of the inventory agencies. An overview of the essential points of the guidelines, citing, step by step, specific UNFCCC decisions or IPCC guidelines chapters is provided in the supplementary material.

## 3 How the research community can contribute to the inventory process

A GHG inventory report includes the common reporting format tables covering all relevant gases, categories and years (see the supplementary materials), and a written report that documents the methodologies and data used to prepare the estimates (dec. 24/CP.19 – UNFCCC, 2014; dec. 18/CMA.1 – UNFCCC, 2019a). The overall reporting process implies the establishment of a national system that includes all institutional, legal and procedural arrangements, including a data management system. Among others, the quality and reliability of the GHG inventories are based on the scientific reliability of the methodologies and models adopted for the calculation of emissions and removals. Inventory compilers are encouraged to continuously improve their estimates, whenever possible, and to build each new inventory on the previous one (IPCC, 2006). In many countries, research institutions are directly involved in the inventory preparation, as they are part of this system providing the estimates for a specific sector/gas (Damassa et al., 2015).

In general, research community efforts can contribute at different levels in the inventory process, such as:

  • providing peer reviewed papers and reports to support inventory compilers to prepare and constantly improve national GHG inventories (Vol.1, Ch.2 – IPCC, 2006);
  • supplying the inventory community with data useful for the verification process performed by the country at each step of the GHG inventory compilation (Vol.1, Ch.1– IPCC, 2006); and
  • developing targeted data and methods that can be used by developing country Parties to fulfil their new reporting obligations.

While the general IPCC and UNFCCC inventory requirements are summarised in the supplementary material, here we focus on the issues that emerged from exchanges between the research community and the inventory agencies within the VERIFY project and from the analysis of existing literature. The main outputs of this process can be summarised in the following main issues: the importance of attribution of GHG emissions and removals to specific and detailed source and sink categories (Section 3.1); what are the main sectors/categories/gases to focus on and the importance of uncertainty analysis (Section 3.2); the issues linked to system boundaries of inventories in terms of spatial and temporal scale (Section 3.3); the understanding of terms and definitions generally used or adopted at country level by the inventory compliers (Section 3.4); the challenges related to land representation (Section 3.5); the inventory verification needs through independent datasets (Section 3.6); and, finally, a focus on the role of research in targeting the emerging needs in developing countries (Section 3.7).

### 3.1 Source and sink attribution

Source/sink sector attribution is a key requisite in the GHG inventory. Five major sectors, covering most emissions and removals, need to be reported in the inventories (dec. 18/CMA.1, par. 50 of the annex – UNFCCC, 2019a):

1) Energy;
2) Industrial Processes and Product Use (IPPU);
3) Agriculture;
4) Land-use, Land-use change and Forestry (LULUCF); and
5) Waste/Wastewater.

Each of these sectors is subdivided into categories and sub-categories (see Table A.1 in supplementary material) and, even more in detail, into processes. Emission categories within sectors can be grouped while it is not possible to group them between sectors. IPCC Guidelines (1996, 2006), and their refinement (IPCC, 2019), provide methodologies for GHG emission/removal estimates for each sector in a specific volume. The only exception is volume 4 “Agriculture, Forestry and Other land Use (AFOLU)” of the 2006 IPCC Guidelines where Agriculture and LULUCF sectors are grouped together. The inventory is constructed from the summation of sectors, categories and sub-categories according to IPCC methodologies.

Often, many top-down methods, such as atmospheric inversion models, while using the bulk changes in atmospheric concentrations, cannot effectively separate the exact source sectors as required by the IPCC Guidelines. This is mainly due to the constraints that models have regarding the scale of atmospheric transport and the sampling of the atmosphere by atmospheric *in-situ* stations or satellites (Peters et al., 2017), though, in some cases, isotopes or co-emitted species can be used to provide some granularity, such as differentiating fossil and biogenic CH_4_ emissions (Saunois et al., 2020). However, the existing inversion systems cannot disaggregate fossil emissions derived from energy and non-energy use of fuels/feedstock (e.g., in the chemical or iron and steel industry) that in the inventories are reported in two separate sectors (in Energy and in IPPU sector, respectively). The top-down methods, therefore, need to be targeted to where they can provide additional information, such as the methane budget to identify the role of fossil, agricultural, and natural sources in the methane fluxes in different coarse regions (Saunois et al., 2020).

The clear attribution of emissions and removals to anthropogenic or natural origin is challenging. According to the UNFCCC framework, GHG inventories should include only anthropogenic emission/removal GHG estimates. Although these fluxes are easy to identify and attribute to Energy, IPPU and Waste sectors, this is not the case for AFOLU. Indeed, large difficulties exist for the identification, attribution and estimation of anthropogenic CO_2_ emissions/removals, especially those related to LULUCF. This is because the land is both a source and a sink of CO_2_ since both natural and anthropogenic drivers can occur simultaneously (IPCC, 2010; Grassi et al., 2018). This is also true for wetlands CH_4_ emissions (Saunois et al., 2020). Clear differentiation of which component is natural and which is anthropogenic has been subject to a long debate (IPCC, 2010). For pragmatic reasons, under the UNFCCC GHG reporting framework, LULUCF anthropogenic fluxes are considered as all those occurring on “managed lands”, defined as “*lands where human interventions and practices have been applied to perform production, ecological or social functions*” (IPCC, 2006). This so-called “*IPCC managed land proxy”* (IPCC, 2010) is far from perfect, because within managed areas it is not possible to separate direct anthropogenic (e.g., land-use change, harvest, etc.) from indirect anthropogenic effects (human-induced environmental changes, including temperature, precipitation, CO_2_ and nitrogen deposition feedbacks) and from natural processes (including climate variability and a background natural disturbance regime). Furthermore, each country has the possibility to set its own definition of managed lands within the broad definition above, based on specific national circumstances. This means that any comparison of anthropogenic emissions/removals among countries can be affected by different management definitions (Ogle et al., 2018). However, the managed land proxy continues to be applied in the 2019 IPCC Refinement, because it is considered the only universally applicable approach within the UNFCCC framework (i.e., usable with all methodological Tiers) to estimate anthropogenic emissions/removals for inventory purposes (Grassi et al., 2018).

A different approach is used in both the IPCC Fifth Assessment report – AR5 – (Ciais et al., 2013; Smith et al., 2014) and the Global Carbon Project (Friedlingstein et al., 2019). These assessments use global bookkeeping models (e.g., Houghton et al., 2012; Hansis et al., 2015), where the LULUCF anthropogenic fluxes include only the emissions/removals derived from direct human-induced activities (i.e., land-use change, harvest). This means that all the other fluxes (indirect anthropogenic and natural ones) occurring on managed lands are not considered anthropogenic and, therefore, they are assigned to the component considered mostly non-anthropogenic (Grassi et al., 2018; Jia et al., 2019; Petrescu et al., 2020), called “residual sink” (IPCC, 2014) or simply “land sink” (Friedlingstein et al., 2019). The difference in global land-related anthropogenic fluxes between bookkeeping models and the summation of country GHG inventories estimates has been quantified around 4–5 Gt CO_2_yr^−1^ (Grassi et al., 2018; Jia et al., 2019), largely attributable to differences in defining what is the anthropogenic land flux. Given the magnitude of these discrepancies, understanding and resolving them is needed to ensure an accurate comparison between global models and country GHG data in the context of the Global Stocktake.

### 3.2 Defining priorities: key categories and uncertainty analysis

Priority for improvement if GHG inventories and research input should focus on key categories, which are those that have a significant influence on a country’s total emissions. The emissions of these categories should be estimated with high methodological complexity approaches (dec. 18/CMA1 – UNFCCC, 2019a). Tiers 2 and 3 (see supplementary material) are considered the most appropriate and accurate methods in general (and specifically for key categories), and where research inputs are most frequently sought by inventory agencies. Tier 3 is the most demanding approach in terms of complexity, spatial and temporal resolution and data requirements (ranging from simple statistical models through to complex ecosystem models). Models are frequently used to assess complex systems (e.g., forest management or F-Gases emissions) and can be used to estimate GHG emission and removal fluxes. However, models are a means of data transformation and do not remove the need for the original data to drive or validate them. In delivering specific models for the inventories, the research community should provide transparent documentation of the validity and completeness of the data, assumptions, equations and models used as it is a critical requirement for all GHG inventories complexity levels (and especially for Tier 3). For future GHG inventories improvements, the 2019 IPCC Refinement provides updated guidance on the use of models for GHG emission/removal estimations (IPCC, 2019 – Vol.1, Ch.6).

**Fig. 1:**
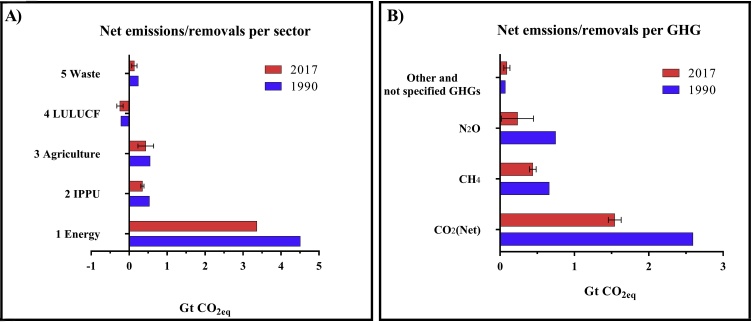
EU 28 (plus Iceland) emissions/removals for the reference year (1990) and 2017 as published in 2019: in Panel A emission (positive values) and removals (negative values) are reported per sector; in Panel B emissions are reported per gas. The bar length represents mean emission/removal values (in Gt CO_2_-eq) while error bars represent the uncertainty. Data source: EEA (2019).

Uncertainty assessment is a fundamental requirement, and it can help the inventory compilers in prioritizing future GHG inventories improvements (Jonas et al., 2010). Large uncertainties hamper progress in designing, implementing and monitoring effective mitigation strategies (Romijn et al., 2018). Generally, rather high uncertainty levels occur when inventory compliers do not have country- or sector-specific data and methodologies and, therefore, they are constrained to applying IPCC default values (Tier 1 level). When country-specific values are available, but there are large uncertainties in the input data, this can lead to uncertainty in emissions estimates. As an example, we report here the case of the EU-27 plus United Kingdom and Iceland (Fig. 1). Although Energy is the sector with the highest total emissions, the estimate of its percentage uncertainty is nearly negligible (1.1 %). Therefore, a greater effort could be focused on refining or improving the estimates of other sectors characterised by higher percentage uncertainties such as Waste, Agriculture, LULUCF and IPPU, where uncertainties are 51.5, 47.0, 34.3 and 11.8 %, respectively (Fig. 1a). Similarly, efforts could be more targeted to reduce the uncertainties of N_2_O emission estimates (even higher than ± 90 %) rather than those for CH_4_ (10.1 %) (Fig. 1b). This does not mean that the reduction of uncertainty for Energy sector is not necessary, but that, in an expected future scenario of GHGs emission reduction from Energy, the proportional role of other sectors and gases could increasingly affect the total relative and absolute uncertainty of emissions estimates for the EU.

Although difficult to assess, developing countries may have different uncertainty reduction needs as non-annex I countries are currently not required to report the uncertainties for their estimates. For example, in China, where well-developed statistical methods have been adopted, the CO_2_ emissions estimates from coal are frequently revised and, often, they contain large anomalies between revisions, thus suggesting high uncertainty in the Energy categories and sub-categories (Korsbakken et al., 2016). LULUCF and Agriculture emissions represent a large portion of many developing countries’ total emissions (Tubiello et al., 2015), thus the uncertainties in those sectors need to be overcome.

Uncertainty is a fundamental measurement for scientific and research outputs, giving inventory compilers a quantitative indication of the reliability of mean estimates and data for assessing inventory uncertainties. When uncertainty measurements are reported, the type of uncertainty methodology used (e.g., standard error or standard deviation of the mean) the number of observations (or replicates) considered needs to be defined. When the data source lacks an uncertainty value or its related information is not clearly defined, inventory compliers are obliged to adopt assumptions for uncertainty estimates which, in turn, add uncertainty to the reliability of the estimates (Carter et al., 2018; Herold et al., 2019).

### 3.3 Temporal and spatial scale

Inventories and scientific studies may operate at different temporal and spatial scales. Therefore, some research studies may not be compatible with the scope (or comparable with the data) of the inventory geographically (Herold et al., 2019) and/or temporally, creating general system boundary problems.

In GHG inventories, the temporal scale is annual. For tracking GHG emissions under the UNFCCC, the results of scientific research must be easy to disaggregate or aggregate into total annual emissions. An example of sub-annual data are those from inverse modelling approaches which are based on continuous concentration data and time-varying transport fields (e.g., Houweling et al., 2015; Bergamaschi et al., 2015, 2018b). Annual aggregation of inverse modelling estimates is not an easy task as uncertainties of flux estimates are temporally correlated and those error correlations must be included when aggregating their output to annual scales. Nonetheless, approaches that operate at sub-annual scale are scientifically useful tools to attribute changes of climate-sensitive emissions to climate events such as droughts and warm winters. For multi-year studies, difficulties may also arise, especially when they do not report clear information regarding the timing of the process described.

In terms of the spatial scale, GHG inventories are based on national territories over which the country has jurisdiction. Exceptions are emissions from fuel use in international maritime and aviation bunkers, which are not allocated to countries. Bunker sales are reported as a “memo items” in national inventories and are not included in the total national GHG emission estimates, yet they can be used as an important information within the Global Stocktake. Although some studies with a more refined spatial scale than inventories may not be compatible with the latter, they could still be useful for country-level estimates (e.g., for new local emission factors - EFs) as long as they are consistent with IPCC guidelines methodologies and their results can be aggregated in a national database. Studies that focus on a multi-national emission/removal GHG footprint should not be considered as a valid inventory inputs unless their results can be properly subdivided according to national boundaries. These results could be included in the set of data useful for verification of some inventories as, for example, those of the European Union (see section 3.6). Similarly, spatial scale problems may arise when the models’ boundaries do not perfectly overlay those of the country. For example, ecosystem models usually fit the ecosystem boundaries which may not overlap with administrative ones.

Sometimes, spatial and temporal scale inconsistencies among research studies and GHG inventories can occur simultaneously. Estimating GHG fluxes of specific gases (like CH_4_ and N_2_O or ozone precursors) at a finer spatial and temporal resolution can be scientifically relevant. For example, some of these gases (CH_4_ and N_2_O) are predominately of microbial origin or have regionally dependent impacts (ozone precursors) and, therefore, they are characterised by high spatial and temporal variability (Leip et al., 2018). Other sources characterised by high temporal variability are related to IPPU. In this sector, the process emissions may vary depending on the operating times and load of installations (e.g., emissions from the chemical industry), and emissions from product use may vary over the year (e.g., more emissions of refrigerants due to operation of air conditioning during summer periods).

Although some detailed scientific studies are too fine in scale to be directly used in inventories, such studies could lead to new fundamental knowledge to better characterise processes. Such information could be used for improving the estimation of activity data and/or emission factors, or for enhancing the accuracy and precision of the models to be used at Tier 3. Conversely, several scientific studies are dependent on different types of data or methods used in the inventories. Inventory agencies and their partners (such as statistical offices) may be well-equipped to provide this information, such as gridded data or sub-annual temporal data, to research communities. There are many potential synergies, for example inventory agencies working at a finer scale than usual, and scientific studies aggregating outputs to coarser scale than usual may both improve data and methods to meet their apparently different objectives.

### 3.4 Terminology and definitions

Different definitions and terms in use can change the scope of what is included in the estimates. This issue is particularly relevant for the LULUCF sector because of the higher system complexity of this sector with respect to the others (Pulles, 2018). Here, definitions of various land-use/land-cover categories (e.g., forest, grassland, wetland etc) can differ among international organisations, such as FAO (Food and Agriculture Organization of the United Nations), Ramsar Convention on Wetlands, and UNFCCC (Federici et al., 2017). For example, forest land definitions are generally based on a list of parameters (e.g., minimum area, minimum plant height at maturity, canopy cover) with different values being applied by different organisations/studies (Federici et al., 2017). The 2006 IPCC Guidelines provide to option to each country to adopt a specific IPCC land-use categories definition to be applied consistently over time, that may or may not aligns with various internationally applied ones (FAO, Ramsar, etc.). Some types of tree cover (like rubber tree or oil palm plantations) can be excluded from the forest definition adopted by the country. These differences can affect the comparability of forest-related emission/removal estimates (Grassi et al., 2017). Full understanding of national definitions in use within the GHG inventory are, therefore, key for providing relevant data inputs.

Considering the land-related GHG fluxes estimated by the research community, Pongratz et al. (2014) pointed out that there are at least nine different published versions of net land-use/land-cover change fluxes’ definitions and related models, the estimates of which differ by a factor of two for the historical period considered. The authors suggested that the problem of global land-use/land-cover change definition and resulting GHG net emission estimates could be solved by disaggregating direct anthropogenic and non-anthropogenic effects in all the possible land-atmosphere fluxes (Gasser and Ciais, 2013; Pongratz et al., 2014). In this context, ideally, inventories and scientific studies should provide sufficient disaggregation of fluxes to allow complete data and definition comparability. Although this problem is not attributable specifically to one of these communities, it may be easier for the scientific studies to provide additional output variables, as the timelines to change reporting guidelines is slow.

### 3.5 Land representation

The representation of land use and land-use change in the GHG inventory is one of the most challenging tasks. The 2006 IPCC Guidelines proposed different approaches at different level of complexity to be applied depending on data availability, ranging from the use of land statistics (e.g., FAOSTAT data) through to a spatially explicit approach (e.g., high-resolution land monitoring techniques). The latter is based on a complete set of data, which allows the total area of each category that has been subjected to any transition to be detected, knowing the precise land-use change (i.e., both the land-use categories before and after the transition) and its location within the national territory (spatially-explicit data) (Vol.4, Ch.3 – IPCC, 2006).

Typically, developed countries have detailed data, such as national forest inventories, available for their GHG inventory estimates. Developing countries, however, have less national statistical data available and their activity data are more frequently based on techniques such as remote sensing data acquisition and elaboration (De Sy et al., 2012; Mitchell et al., 2017). The 2019 IPCC Refinement emphasises the increasing potential of remote sensing products for LULUCF emission/removal estimates. Although their potential value is high, there are several challenges to overcome in their use for inventory reporting activities. These challenges are mainly related to spatial resolution (that should be consistent with land category definitions used by the countries under the UNFCCC); the geographic extent (that needs to be national); temporal coverage (that needs to be consistent along the time series) and definitions in use (Romijn et al., 2018; Herold et al., 2019). Satellite data are mostly appropriate in land cover and land-cover change classification (i.e., area of tree cover, deforestation), while IPCC and UNFCCC focus on land use and land management (i.e., areas of managed forest) to effectively track the anthropogenic components of the GHG fluxes (GFOI, 2016; Romijn et al., 2018). Generally, it is difficult to identify specific changes in management practices within forest, cropland and grassland from satellite data, which can affect the carbon stock change estimates reported in inventories. Conversely, satellite data can indicate a change in forest land cover over a harvested patch, while a no real land- use change occurred. This problem highlights the importance of having an integrated monitoring system, based in part on the acquisition and interpretation of satellite images, and in part on national scale statistical data derived from field surveys (Szantoi et al., 2020). This makes it possible to reclassify land cover products into land use categories. Furthermore, incorporating remote sensed data in the national GHG inventory requires a long-term perspective, such as establishing a data protocol to assure accessible data in the future and their consistency across time-series (Herold et al., 2019).

### 3.6 GHG inventory verification

The main purpose of verification activities is to provide information on how a country’s GHG inventory can be improved. Verification is addressed at international level through the review processes under the UNFCCC and Paris Agreement, to support transparency of information provided, and domestically, as part of the national GHG monitoring, reporting and verification system. The domestic verification is performed within the inventory compilation by the inventory agencies, and it is based on external methods using independent datasets. The 2006 IPCC Guidelines include examples of institutions that provide public data that may be useful for this purpose (e.g., FAOSTAT^2^, EUROSTAT^3^ and EDGAR^4^). National estimates derived from independent sources and based on different methods can be compared to the GHG inventory within individual sectors. This type of comparison helps to identify major calculation errors or may highlight a subcategory in any sector that has been omitted or falsely allocated in the calculations.

By comparing inventory estimates to independent data, it may highlight significant differences which could be associated to either or both methods used. The validity of the verification process must be based on the agreed interpretation of the terminology and definitions adopted by the independent data providers and by the inventory compliers (see section 3.4). IPCC (2014), Tubiello et al. (2015) and Jia et al. (2019) compared CO_2_-eq emissions within AFOLU using EDGAR and FAOSTAT (Agriculture and LULUCF) datasets, EPA^5^ (Agriculture only), and the bookkeeping model by Houghton et al. (2012) (LULUCF). For Agriculture, the datasets were similar. For land-related CO_2_ emissions from LULUCF, while the results from all three datasets and methods were within the same order of magnitude, those based on EDGAR dataset exhibited higher emissions and different trends (IPCC, 2014). This is because EDGAR included only a dataset on fire and decay emissions, which does not reflect the complexity of LULUCF carbon fluxes. For this reason, more recent versions of EDGAR (from v4.3.2 onwards) no longer include these land-related CO_2_ estimates, which will be updated in future versions when greater comparability with country-based LULUCF fluxes can be ensured. Andrew (2020) performed a comparison of CO_2_ emissions datasets from fossil carbon sources finding significant deviation between estimates, mainly attributable to system boundaries. The author concluded that greater effort in improving transparency and data disaggregation is essential to understand and reconcile differences between independent datasets as well as between each dataset and the inventories. This is true whether the independent datasets are used to improve inventories, for domestic verification, or as part of the Global Stocktake.

The 2006 IPCC Guidelines pointed out that “*National GHG inventory estimates from Tier 3 models can be difficult to verify because alternative measurements often do not exist at the national level [for the whole AFOLU sector] … there may however be opportunities to verify component estimates against independent data*”. The 2019 IPCC Refinement highlights an additional verification method: “*An ideal condition for verification is the use of fully independent data as a basis for comparison. Measurements of atmospheric concentrations provide such datasets, and recent scientific advances allow using such data as a basis for emission modeling.*” These are generally known as atmospheric inversion models or top-down models, which are considered as promising approaches to validate not only emission estimates from Energy and IPPU (sectors where they are already widely used), but also those from Agriculture.

Atmospheric inversion techniques have proved to provide useful information for the national budgets of non−CO_2_ gases like CH_4_ or some F-gases where data coverage is sufficient (Ganesan et al., 2017; Rigby et al., 2019). UK (Manning et al., 2011), Switzerland, Australia and New Zealand have used these techniques as part of their inventory verification process. Fossil CO_2_ has a different carbon isotopic signature with respect to that of other fluxes. Therefore, it can be used, in principle, to attribute emissions to specific sources, although these multi-tracer inversions are still at their infancy. However, these models are problematic for LULUCF sector as they cannot distinguish the anthropogenic and natural CO_2_ fluxes that can occur simultaneously (Vol.1, Ch.6 – IPCC, 2019). Examples of these applications are the works of Manning et al. (2011) and Bergamaschi et al. (2018b) for N_2_O emissions by agricultural soils, and of Ganesan et al. (2017) for both N_2_O and CH_4_. Inversion methods have also been used to detect underreporting of HFC in Europe (e.g., Keller et al., 2012; Maione et al., 2014) and China (e.g., Stanley et al., 2020), and of CH_4_ in the United States (Miller et al., 2013).

One way to make inversions more directly relevant to inventory improvement needs is to compute emission factors (EF) from their output, which can be directly compared with values used by countries. Examples of EFs retrieved by dividing top-down flux estimates are the recent studies of Miller et al. (2019), who have estimated higher EF for coal mining CH_4_ emissions in China than inventories; and of Thompson et al. (2019), who have estimated a higher global EF for N_2_O emissions than IPCC Tier 1 for Agriculture.

The main limitations to the application of inversion models for verification purposes are linked to system boundaries and source attribution (see Sections 3.1 and 3.3). For example, air flow crosses national borders may make it difficult to separate national contributions by inverse modelling technique (Vol.1, Ch.6 – IPCC, 2019) in areas with high emission densities from multiple countries. Overcoming such an obstacle may be possible with a dense network of atmospheric observations (from *in-situ* and/or remote sensing), which is able to separate out a ‘dirty’ variable background airflow coming from a neighbouring country. Alternatively, atmospheric data from multiple countries could be aggregated and verified at the continent (e.g., EU) level rather than the country level.

Independent reference data from the scientific community (e.g., Harris et al., 2021) may provide an important source of information for GHG emissions/removals, helping to increase transparency and accuracy of the reporting system, and mitigation framework under the Paris Agreement. To be effective and useful, data need to be as comparable as possible with countries’ definitions and levels of aggregation (Grassi et al., 2018), be free and open access, and accompanied by a transparent description of data, methods, assumptions and uncertainties (Romijn et al., 2018; López-Ballesteros et al., 2020).

### 3.7 Emerging needs in developing countries

The Paris Agreement will pose new challenges to developing countries that will need to establish inventory institutional and data management systems. At present, many of these Parties do not yet have the necessary capacity in place. Ability to regularly report national GHG inventories varies among Parties and it is closely related to the efforts and achievements of previous inventory preparations (Umemiya et al., 2017). So far, 63 developing countries have submitted their first Biannual Update Report (BUR) under UNFCCC, 31 the second, 12 the third and only 2 the fourth one, with 91 countries that have not submitted any yet. Meeting the reporting requirements requires improvements in the availability of basic technical capacity, such as statistics and the scientific expertise. This demands, among others, personnel training, institutional funding, establishment of a network for the involved institutions, definition of procedures and responsibility and, therefore, a system that is backed by a strong regulatory framework (Damassa and Elsayed, 2013; Umemiya et al., 2017; Kawanishi and Fujikura, 2018). Although very little can be done by the research community to address the institutional framework, capacities and funding needs, there is a great potential in supporting the development of GHG monitoring systems through different pathways. Contributions can be made by: promoting standardised methodological protocols for data gathering and tools for data elaboration; publishing new findings for the refinement of emission factors and tools for the gathering activity data; and, guaranteeing the availability and accessibility to the existing methodological know-how that is crucial to fill existing observational gaps (López-Ballesteros et al., 2020). Development of facilitative software could also help in preparing and managing GHG inventories (Tulyasuwan et al., 2012; López-Ballesteros et al., 2020). While research needs may vary from country to country, Agriculture and LULUCF sectors are broadly considered the GHG emission hotspots in developing countries, where such sectors play a significant role as they represent a large portion of both national economies and total emissions (Tubiello et al., 2015; Baccini et al., 2017; Peters et al., 2017). On average in developing countries, Agriculture emissions contribute 35 % of national emissions (up to 50 % in low income developing countries), whereas they contribute 12 % in developed countries (Richards et al., 2015). Tongwane and Moeletsi (2018) estimated that the GHG emissions from agriculture in Africa increased at an average annual rate of around 3% between 1994 and 2014. Tian et al. (2020) indicated that Brazil, China and India have driven the global N_2_O emissions increase (mainly) from the Agriculture sector, over the most recent decade. Regarding the LULUCF sector, the last Forest Resource Assessment (FAO, 2020) identifies Africa and South America as the (sub-)continents with the highest annual rate of net forest loss since 1990. Developing countries in all regions, particularly the least-developed ones, have put a strong emphasis on the mitigation potential of Agriculture and/or LULUCF in their NDCs, highlighting also the vulnerabilities of these sectors to climate change (FAO, 2016). Agriculture and LULUCF sectors are the most complex, expensive, time consuming to measure, and the most difficult to monitor. For these sectors, the use of tier 1 methodologies in the GHG Inventory reporting is widespread due to a severe lack of measured data, often accompanied by limited technical capacities (Tulyasuwan et al., 2012; Pelster et al., 2016; Goopy et al., 2018; Pulles, 2018; Tian et al., 2020).

A good example of fruitful exchange between science, technical and policy communities in the AFOLU sectors are the methods and guidance documents of the Global Forestry Observation Initiative (GFOI) that provides methodological advice and assistance on forest monitoring and reporting systems in line with IPCC Guidelines. GFOI also makes data and methods available for using remotely sensed and ground-based data, thus becoming an important reference for many REDD + countries (GFOI, 2020).

To be effectively useful for inventory reporting in developing countries, any methods, protocol, tool and research infrastructure should consider related costs, maintenance effort and knowledge needed, which can vary from country to country. Overall, a careful assessment with the end users of what can be effectively developed and maintained according to the country’s circumstances and operational environment is key to providing a tool that meets user requirements in terms of practical and operational implementation (Ochieng et al., 2016; Herold et al., 2019).

Alongside methods and data, financial support dedicated to fund country-specific research activities and training of GHG management personnel is very beneficial for improving inventory capacity (Damassa and Elsayed, 2013). Finally, cooperative programs are useful to develop/improve regional activity data sharing among countries with similar biogeographical backgrounds or aiming to exchange audits as part of quality assurance activities (Tulyasuwan et al., 2012).

## 4 Conclusion

The research community already plays a key role in increasing confidence in country GHG estimates, which are the basis of any climate policy. However, emerging challenges under the Paris Agreement require this community to make additional efforts to support the process at different levels, which span from the improvement of country GHG inventory estimates and their verification (particularly in developing countries) to the assessment of the collective climate change mitigation progress within the Global Stocktake. To effectively contribute, the research community needs to better understand terms, rules, procedures and guidelines that countries follow to estimate and report their GHG emissions under the Paris Agreement. Too often, scientific papers speak a language which is different from that used by the GHG inventory community. The LULUCF sector is a good example of this communication challenge. Due to the high level of complexity of its carbon dynamics, and the difficulty of differentiating the anthropogenic and non-anthropogenic components, the research community and the GHG inventory community have developed different approaches to estimate anthropogenic land-related fluxes. Each approach has its own advantages and limitations – the real problem is that they are not fully comparable. Reconciling these differences does not require that the research community abandon its own approach, but rather that solutions are found to ensure comparability.

To be relevant for the improvement of countries’ GHG inventories, research products should provide: clear and transparent definitions of terminology and attribution to processes or sub-sectors; detailed information on methodologies and uncertainties of estimations; data and results at suitable scales (national/annual) whilst taking into consideration, as far as possible, the glossary of terms and categorisations defined by the IPCC inventory community. Clarity with measurement units and sticking to original mass units of individual GHG where possible and providing geographical coordinates (if spatial data are included) are also key. Estimates compatible with the IPCC sectors and categories can better serve the needs of the GHG inventory community. Clear declaration of possible categories that are covered by the measurement will help to increase the understanding of the components included. Similarly, the 2019 IPCC Refinement calls for greater levels of transparency and disaggregation by inventory compilers where possible (IPCC, 2019). Consultations between relevant experts from the inventory community and scientific researchers can support attribution of categories with the related geographic area/sector of interest.

Developing country Parties face new reporting obligations in conditions often affected by poor capacities and lack of data availability. Tools and methods that assist these countries to effectively address this challenge are of utmost importance, but specific attention should be paid to the country/region needs, consideration of related costs, maintenance effort of tools and knowledge needed. Priority efforts should be focused on LULUCF and Agriculture sectors compared to the others, as they are the most complex to measure, have high associated uncertainties, and, at the same time, are the most important sectors in terms of contribution to total emissions for many developing countries. The policy process would greatly benefit from science that considers specific inventory needs, and is targeted for the different global regions that provides data and knowledge (i.e., methodological guidance and research results) through free access to well documented independent databases, research infrastructures and shared protocols for data gathering. Conversely, GHG inventories can represent a valid source of data that is constantly reviewed and updated and that can be particularly useful for research studies. Therefore, promotion of national and regional networking initiatives on specific topics can help both communities in exchanging of data and methods, solving interpretative problems, and understanding each other's data and needs.

Actively involving the inventory community within research projects is a valid option for an enhanced exchange and efficient provision of data and information between the two communities and should be further promoted by national and international research funders.

## Contributions

**Lucia Perugini**: Conceptualization, Methodology, Investigation, Writing - Original Draft, Supervision; **Guido Pellis**: Conceptualization, Methodology, Formal analysis, Investigation, Writing - Original Draft; **Giacomo Grassi**: Writing - Review and Editing, Resources; **Philippe Ciais**: Writing - Review and Editing; **Han Dolman**: Writing - Review and Editing; **Joanna I. House**: Writing - Original Draft, Review and Editing, Resources; **Glen Peters**: Writing - Review and Editing, Resources; **Pete Smith**: Writing - Review and Editing; **Dirk Günther**: Writing - Review and Editing, Supervision; **Philippe Peylin**: Writing - Review and Editing, Supervision, Resources, Funding acquisition.

## Declaration of Competing Interest

The authors report no declarations of interest.

## Footnotes

1 https://verify.lsce.ipsl.fr/

2 http://www.fao.org/faostat/en/

3 https://ec.europa.eu/eurostat

4 https://edgar.jrc.ec.europa.eu/

5 https://enviro.epa.gov/
