# Supplementary material for: Emerging reporting and verification needs under the Paris Agreement: How can the research community effectively contribute?
Source: Environ Sci Policy. 2021 Aug;122:116–26. doi: 10.1016/j.envsci.2021.04.012 (PMC8171125; doi:10.1016/j.envsci.2021.04.012)
Supplement: Supplementary file 2 [file mmc2.docx]

# SUPPLEMENTARY MATERIAL

# Reporting requirements and GHG inventories under the Enhanced Transparency Framework

There are five general reporting principles under the UNFCCC (dec. 24/CP.19, par. 4 of annex I – UNFCCC, 2014) and the Paris Agreement (art.4.13 – UNFCCC, 2015; dec. 18/CMA.1, par. 3(d) of the annex – UNFCCC, 2019a) to ensure improving quality of the inventories (and NDCs) over time:

- *Transparency*, that refers to clear and sufficient documentation selected for data sources and assumptions, and for the adopted methodologies;
- *Completeness* of the GHG inventory that should include all gasses emitted/removed by all sources/sinks under the national jurisdiction (details are given below) for all the years of the time-series;
- *Consistency* of the data selection and methodological choices adopted for all the years of the time-series;
- *Comparability*, that refers to the application of methodologies and formats (e.g., common reporting tables) that allow simple and credible emission and removal estimates comparison among Parties’ GHG inventories;
- *Accuracy* of all emissions/removals, which should neither over- nor under-estimate.

As the ultimate objective of the UNFCCC framework is to limit the anthropogenic interference with the climate system, the scope of the inventory is focused on the emissions and removals of GHGs that are human induced and not already covered by the Montreal Protocol for ozone depleting substances. Countries shall report the anthropogenic emissions and removals following a source-based principle: identifying the source from which the emissions originated, and the sink where the GHGs are removed. Five major sectors**,** covering most emissions and removals, need to be reported (dec. 18/CMA.1, par. 50 of the annex – UNFCCC, 2019a):

1. Energy;
2. Industrial Processes and Product Use (IPPU);
3. Agriculture;
4. Land-use, Land-use change and Forestry (LULUCF); and
5. Waste/Wastewater.

Each of these sectors is subdivided into categories and sub-categories (see Table A.1 at the end of the supplementary material) and, even more in detail, into processes. IPCC Guidelines (1996, 2006), and their refinement (IPCC, 2019), provide methodologies for GHG emission/removal estimates for each sector in a specific volume. The only one exception is volume 4 Agriculture, Forestry and Other land Use (AFOLU), of the IPCC 2006 guidelines where Agriculture and LULUCF sectors are grouped together.

Emissions and removals for all GHGs and carbon pools listed in the 2006 IPCC Guidelines should be estimated by Parties at country level on annual basis from 1990 to the submission year minus 2 (as time series), or minus 3 for developing countries that need flexibility. To ensure time series consistency, the same methods and approaches should be applied throughout the time series (i.e., if a method is updated, the whole time series must be recalculated). The full time series shall be submitted in the biannual national inventory report. Developing countries are allowed some flexibility on the starting date of reporting, although it is compulsory to include the same base year/period that is used in the country NDC and the estimates from 2020 onward (dec. 18/CMA.1, par. 57 of the annex – UNFCCC, 2019a).

Seven GHGs must be reported by all Parties: carbon dioxide, (CO_2_); methane (CH_4_), nitrous oxide (N_2_O) and the so-called F-gases, which are hydrofluorocarbons (HFCs), perfluorocarbons (PFCs), sulfur hexafluoride (SF_6_) and nitrogen trifluoride (NF_3_). When HFCs, PFCs, SF_6_ and NF_3_ are reported, they must be disaggregated into their specific chemicals (e.g., HFC-23). Developing countries that need it, in the light of their capacities, have the flexibility to limit their reporting to the most important gases (CO_2_, CH_4_ and N_2_O) as well as any of the additional gas that is included in their NDC or in any previous GHG inventory. For the inventory purposes, all the GHGs have to be reported in a mass unit, which is generally metric tonnes or its multiples, or Giga grams.

The different GHGs cannot be added together unless they are transformed to a comparable metric. In GHG inventories, this has been done using Global Warming Potentials (GWPs), though it is noted that there is still considerable scientific debate regarding these emission metrics (e.g., Myhre et al*.*, 2013; IPCC, 2014; Harmsen et al., 2016; Allen et al., 2018; Cain et al., 2019). GWP is the comparison (i.e., ratio) of the cumulative radiative forcing of one unit of mass of a specific GHG, relative to the same mass of CO_2_ over a specific time period. The Katowice Rulebook establishes that, for GHG inventory purposes, the GWPs with a 100-year-time-horizion from the IPCC fifth assessment report shall be used (IPCC, 2014), or any 100-year-time-horizon GWP value from the subsequent agreed IPCC assessment reports if agreed by Parties of the Paris Agreement. These metrics will be used to express the aggregated emissions and removals in CO_2_ equivalent (CO_2_-eq).

Each Party should also provide information on precursors of GHGs and indirect emissions. Precursors are gasses not included in the GWP-weighted GHGs emission totals but, when released into the atmosphere, they contribute to the formation of GHGs previously listed (e.g., CO_2_) or other GHGs (e.g., ozone – O_3_), or other molecules which play a role in the climate change (e.g., sulphate particles) (Vol.1, Ch.7 – IPCC, 2019a). The precursors of GHGs or of molecules that play a role in climate change that should be included in the emission estimates are carbon monoxide (CO), nitrogen oxides (NO_x_), non-methane volatile organic compounds (NMVOCs) and sulphur dioxide (SO_2_). According to 2006 IPCC Guidelines (Vol.1, Ch.7), the indirect emissions that should be reported are all N_2_O emissions that cannot be *directly attributed* to human activities. This means that N_2_O emissions caused by human-induced nitrogen input to the soil as, for example, agricultural management practices, have to be considered as direct emissions. On the other hand, the indirect N_2_O emissions are only those caused by atmospheric deposition of nitrogen in NOx and NH_3_, and nitrogen surfaces runoff and leaching (Vol.1, Ch.7 – IPCC, 2006). According to the Katowice Rulebook (2019a, dec. 18/CMA.1, par. 52 of the annex), indirect N_2_O emissions related to sectors other than Agriculture shall be reported as “memo items”. In addition, according to the same source, indirect CO_2_ emission caused by atmospheric oxidation of CH_4_, CO and NMVOCs can be included, and if they are, then national totals should be reported with and without them.

The basic methodological approach of GHG inventories estimates consists in the combination of the information on the extent to which a human activity takes place (i.e., activity data – AD) with coefficients that quantify the emissions or removals per unit of activity (i.e., emission factors – EF) according to the following formula:

$$Emissions=AD\cdot EF$$

This simple equation can, in some circumstances, be modified to include other relevant parameters or to better estimate the emissions by complex modelling approaches (IPCC, 2006 – Vol.1, Ch.1). The estimates complexity level of the methodology applied is organized into three tiers. Tier 1 methods represent the lowest level of complexity and are provided for all categories by IPCC Guidelines. They are designed to use readily available national or international statistics to define the AD, in combination with default EF provided by the IPCC Emission Factor Database^[[1]](#footnote-1)^. Tier 2 follows the same method as Tier 1 but uses country- or region-specific data/EF. Tier 3 is the most demanding one in terms of complexity, spatial and temporal resolution and data requirements (ranging from simple statistical models through to complex ecosystem models). Tiers 2 and 3 are considered to be the most appropriate and accurate methods in general, while Tier 1 allows all parties to estimate their emissions even when country specific emission factors are not available. Higher tiers (Tier 2 and Tier 3) are required for those categories that have a significant influence on a country’s total inventory of GHGs, so called “key categories” (dec. 18/CMA.1, par. 25 of the annex – UNFCCC, 2019a; Vol.1, Ch.4 – IPCC, 2019a). As defined in the Katowice Rulebook, developing countries have some flexibility to identify the key categories, on the basis of their capacities.

GHG emission/removal estimates are inherently characterised by uncertainties that need to be assessed and quantified for each category and for the total inventory (both including and excluding LULUCF sector). Uncertainty assessment is extremely important to identify both the priority to reduce the total inventory uncertainty and to drive decision for future methodological choices. Uncertainties depend on the analyst’s state of knowledge, which, in turn, depends on the quality and quantity of applicable data as well as knowledge of underlying processes and inference methods. The quantitative uncertainty analysis is performed by estimating the 95 percent confidence interval of the GHG emissions/removals for the individual gas, process, category, sector, as well for the total inventory. The 2006 IPCC Guidelines suggest two approaches to estimate uncertainty that are described in detail in volume 1, chapter 3 of the guidelines.

Following the 2006 IPCC Guidelines, during the inventory process, inventory compilers are also encouraged to follow the good practice of verifying their data and results against independent science-based estimates, such as peer-reviewed papers (Vol.1, Ch.6 – IPCC, 2006).

**Table A.1 – Main subdivision of inventory sectors. Each sector is disaggregated into categories and sub-categories. The subdivision and the codes follow the structure of the common reporting tables (CRT) currently used by Annex I Parties under the current reporting framework.**

| Sector code | Sector name | Category code | Category name | Sub-category code | | Sub-category name |  |
| --- | --- | --- | --- | --- | --- | --- | --- |
| 1 | **ENERGY** | **1.A** | **Fuel Combustion Activity** | **1.A.1** | | **Energy Industries** |  |
|  |  |  |  | **1.A.2** | | **Manufacturing industries and construction** |  |
|  |  |  |  | **1.A.3** | | **Transport** |  |
|  |  |  |  | **1.A.4** | | **Other Sectors** |  |
|  |  |  |  | **1.A.5** | | **Other - Military** |  |
|  |  | **1.B** | **Fugitive Emission from Fuels** | **1.B.1** | | **Solid Fuels** |  |
|  |  |  |  | **1.B.2** | | **Oil and Natural Gas** |  |
|  |  | **1.C** | **Carbon Dioxide Transport and Storage** | **1.C.1** | | **Transport of CO_2_** |  |
|  |  |  |  | **1.C.2** | | **Injection and Storage** |  |
|  |  |  |  | **1.C.3** | | **Other** |  |
|  |  | **1.D** | **International transport** |  | | |  |
| 2 | **INDUSTRIAL PROCESS AND PRODUCT USE (IPPU)** | **2.A** | **Mineral Industry** | **2.A.1** | **Cement production** | |  |
|  |  |  |  | **2.A.2** | **Lime Production** | |  |
|  |  |  |  | **2.A.3** | **Glass Production** | |  |
|  |  |  |  | **2.A.4** | **Other Process Uses of Carbonates** | |  |
|  |  |  |  | **2.A.5** | **Other** | |  |
|  |  | **2.B** | **Chemical Industry** | **2.B.1** | **Ammonia Production** | |  |
|  |  |  |  | **2.B.2** | **Nitric Acid Production** | |  |
|  |  |  |  | **2.B.3** | **Adipic Acid Production** | |  |
|  |  |  |  | **2.B.4** | **Caprolactam, Glyoxal and Glyoxylic Acid Production** | |  |
|  |  |  |  | **2.B.5** | **Carbide Production** | |  |
|  |  |  |  | **2.B.6** | **Titanium Dioxide Production** | |  |
|  |  |  |  | **2.B.7** | **Soda Ash Production** | |  |
|  |  |  |  | **2.B.8** | **Petrochemical and Carbon Black Production** | |  |
|  |  |  |  | **2.B.9** | **Fluorochemical Production** | |  |
|  |  |  |  | **2.B.10** | **Other (Dodecandioic acid and fertilizer)** | |  |
|  |  | **2.C** | **Metal Industry** | **2.C.1** | **Iron and Steel Production** | |  |
|  |  |  |  | **2.C.2** | **Ferroalloys Production** | |  |
|  |  |  |  | **2.C.3** | **Aluminium Production** | |  |
|  |  |  |  | **2.C.4** | **Magnesium Production** | |  |
|  |  |  |  | **2.C.5** | **Lead Production** | |  |
|  |  |  |  | **2.C.6** | **Zinc Production** | |  |
|  |  |  |  | **2.C.7** | **Other (Copper)** | |  |
|  |  | **2.D** | **Non-Energy Products from Fuels and Solvent Use** | **2.D.1** | **Lubricant Use** | |  |
|  |  |  |  | **2.D.2** | **Paraffin Wax Use** | |  |
|  |  |  |  | **2.D.3** | **Other** | |  |
|  |  | **2.E** | **Electronics Industry** | **2.E.1** | **Integrated Circuit or Semiconductor** | |  |
|  |  |  |  | **2.E.2** | **TFT Flat Panel Display** | |  |
|  |  |  |  | **2.E.3** | **Photovoltaics** | |  |
|  |  |  |  | **2.E.4** | **Heat Transfer Fluid** | |  |
|  |  |  |  | **2.E.5** | **Other** | |  |
|  |  | **2.F** | **Product Uses as Substitutes for Ozone Depleting Substances** | **2.F.1** | **Refrigeration and Air Conditioning** | |  |
|  |  |  |  | **2.F.2** | **Foam Blowing Agents** | |  |
|  |  |  |  | **2.F.3** | **Fire Protection** | |  |
|  |  |  |  | **2.F.4** | **Aerosols** | |  |
|  |  |  |  | **2.F.5** | **Solvents** | |  |
|  |  |  |  | **2.F.6** | **Other Applications** | |  |
|  |  | **2.G** | **Other product manufacture and use** | **2.G.1** | **Electrical Equipment** | |  |
|  |  |  |  | **2.G.2** | **SF_6_ and PFCs from Other Product Uses** | |  |
|  |  |  |  | **2.G.3** | **Medical application and N_2_O from Product Uses** | |  |
|  |  |  |  | **2.G.4** | **Other** | |  |
|  |  | **2.H** | **Other** | **2.H.1** | **Pulp and Paper Industry** | |  |
|  |  |  |  | **2.H.2** | **Food and Beverages Industry** | |  |
|  |  |  |  | **2.H.3** | **Other** | |  |
| 3 | **AGRICULTURE** | **3.A** | **Enteric fermentation** | **3.A.1** | **Cattle** | |  |
|  |  |  |  | **3.A.2** | **Sheep** | |  |
|  |  |  |  | **3.A.3** | **Swine** | |  |
|  |  |  |  | **3.A.4** | **Other livestock** | |  |
|  |  | **3.B** | **Manure management** | **3.B.1** | **Cattle** | |  |
|  |  |  |  | **3.B.2** | **Sheep** | |  |
|  |  |  |  | **3.B.3** | **Swine** | |  |
|  |  |  |  | **3.B.4** | **Other livestock** | |  |
|  |  | **3.C** | **Rice cultivation** | **3.C.1** | **Irrigated** | |  |
|  |  |  |  | **3.C.2** | **Rainfed** | |  |
|  |  |  |  | **3.C.3** | **Deep water** | |  |
|  |  |  |  | **3.C.4** | **Other** | |  |
|  |  | **3.D** | **Agricultural soils** | **3.D (a)** | **Direct N_2_O emissions from managed soils** | |  |
|  |  |  |  | **3.D (b)** | **Indirect N_2_O Emissions from managed soils** | |  |
|  |  | **3.E** | **Prescribed burning of savannas** |  | | |  |
|  |  | **3.F** | **Field burning of agricultural residues** | **3 F 1** | **Cereals** | |  |
|  |  |  |  | **3 F 2** | **Pulses** | |  |
|  |  |  |  | **3 F 3** | **Tubers and roots** | |  |
|  |  |  |  | **3 F 4** | **Sugar cane** | |  |
|  |  |  |  | **3 F 5** | **Other** | |  |
|  |  | **3.G** | **Liming** |  | | |  |
|  |  | **3.H** | **Urea application** |  | | |  |
|  |  | **3.I** | **Other carbon containing fertilizers** |  | | |  |
|  |  | **3.J** | **Other** |  | | |  |
| **LULUCF** | | **4.I** | **Direct N_2_O emissions from nitrogen inputs to managed soil** |  | | |  |
|  |  | **4.II** | **Emissions and removals from drainage and rewetting and other management of organic and mineral soils** |  | | |  |
|  |  | **4.III** | **Direct N_2_O emissions from nitrogen mineralization/immobilization associated with loss/gain of soil organic matter resulting from change of land use or management of mineral soils** |  | | |  |
|  |  | **4.IV** | **Indirect nitrous oxide (N_2_O) emissions from managed soils** |  | | |  |
|  |  | **4.V** | **Biomass Burning** |  | | |  |
|  |  | **4.A** | **Forest Land** | **4.A (a)** | Soil, Litter, Dead organic matter | |  |
|  |  |  |  | **4.A (b)** | Above and below ground biomass | | |
|  |  | **4.B** | **Cropland** |  | | |  |
|  |  | **4.C** | **Grassland** |  | | |  |
|  |  | **4.D** | **Wetlands** |  | | |  |
|  |  | **4.E** | **Settlements** |  | | |  |
|  |  | **4.F** | **Other Land** |  | | |  |
|  |  | **4.G** | **Harvested Wood Products** |  | | |  |
|  |  | **4.H** | **Other** |  | | |  |
| 5 | **WASTE** | **5.A** | **Solid Waste Disposal** | **5.A.1** | **Managed Waste Disposal Sites** | |  |
|  |  |  |  | **5.A.2** | **Unmanaged Waste Disposal Sites** | |  |
|  |  |  |  | **5.A.3** | **Uncategorised Waste Disposal Sites** | |  |
|  |  | **5.B** | **Biological Treatment of Solid Waste** | **5.B.1** | **Composting** | |  |
|  |  |  |  | **5.B.2** | **Anaerobic digestion at biogas facilities** | |  |
|  |  | **5.C** | **Incineration and Open Burning of Waste** | **5.C.1** | Waste Incineration | |  |
|  |  |  |  | **5.C.2** | **Open Burning of Waste** | |  |
|  |  | **5.D** | **Wastewater Treatment and Discharge** | **5.D.1** | **Domestic Wastewater Treatment and Discharge** | |  |
|  |  |  |  | **5.D.2** | **Industrial Wastewater Treatment and Discharge** | |  |
|  |  |  |  | **5.D.3** | **Other** | |  |
|  |  | **5.E** | **Other (please specify)** |  | | |  |

# References

Allen, M. R., Shine, K. P., Fuglestvedt, J. S., Millar, R. J., Cain, M., Frame, D. J., Macey, A. H., 2018. A solution to the misrepresentations of CO_2_-equivalent emissions of short-lived climate pollutants under ambitious mitigation. *npj Climate and Atmospheric Science*. 1, 16. https://doi.org/10.1038/s41612-018-0026-8.

Cain, M., Lynch, J., Allen, M. R., Fuglestvedt J. S., Frame, D. J., Macey, A. H., 2019. Improved calculation of warming-equivalent emissions for short-lived climate pollutants. *npj Climate and Atmospheric Science*. 2, 29. https://doi.org/10.1038/s41612-019-0086-4.

Harmsen, M. J. H. M., van den Berg, M., Krey, V., Luderer, G., Marcucci, A., Stefler, J., Van Vuuren, D. P., 2016. How climate metrics affect global mitigation strategies and costs: a multi-model study. *Climatic Change*. 136(2), 203-216. https://doi.org/10.1007/s10584-016-1603-7.

Myhre, G., Shindell, D., Bréon, F.-M., Collins, W., Fuglestvedt, J., Huang, J., Koch, D., Lamarque, J.-F., Lee, D., Mendoza, B., Nakajima, T., Robock, A., Stephens, G., Takemura, T., Zhang, H., 2013. Anthropogenic and Natural Radiative Forcing. In: *Climate Change 2013: The Physical Science Basis. Contribution of Working Group I to the Fifth Assessment Report of the Intergovernmental Panel on Climate Change* [Stocker, T.F., D. Qin, G.-K. Plattner, M. Tignor, S.K. Allen, J. Boschung, A. Nauels, Y. Xia, V. Bex, P.M. Midgley (eds.)]. Cambridge University Press, Cambridge, United Kingdom and New York, NY, USA.

1. <https://www.ipcc-nggip.iges.or.jp/EFDB/main.php> [↑](#footnote-ref-1)
